# Surface Oxygen Vacancy Modulation of Nanostructured Li-Rich Mn-Based Oxides for Lithium-Ion Batteries

**DOI:** 10.3390/ma18112537

**Published:** 2025-05-28

**Authors:** Jinxia Nong, Xiayan Zhao, Fangan Liang, Shengkun Jia, Zhengguang Zou

**Affiliations:** College of Materials Science and Engineering, Guilin University of Technology, Guilin 541004, China; zhbnxhz@163.com (J.N.); zhaoxy0416@163.com (X.Z.); planliang029@163.com (F.L.); 15670958087@163.com (S.J.)

**Keywords:** lithium-ion battery, Li-rich Mn-based oxides, oxygen vacancies, spinel phase, first principles calculations, electrochemical performance

## Abstract

Li-rich Mn-based cathode materials are considered potential cathode materials for next-generation lithium-ion batteries due to their outstanding theoretical capacity and energy density. Nonetheless, challenges like oxygen loss, transition metal migration, and structural changes during cycling have limited their potential for commercialization. The work in this study employed a straightforward heat treatment to generate oxygen vacancies. This process led to the development of a spinel phase on the surface, which improved Li^+^ diffusion and boosted the electrochemical performance of Li-rich Mn-based oxides. The results demonstrate that the treated Li_1.2_Mn_0.54_Ni_0.13_Co_0.13_O_2_ exhibits an initial specific capacity of 247 mAh·g^−1^ at 0.2C, as well as a reversible capacity of 224 mAh·g^−1^ after 100 cycles, with a capacity retention of 90.7%. The voltage decay is 1.221 mV per cycle under 1C long-term cycling conditions, indicating excellent cycling stability and minimal voltage drop. Therefore, this strategy of engineering through nanoscale oxygen vacancies provides a new idea for the development of high-stability layered oxide anodes and provides a reference for the development and application of new energy materials.

## 1. Introduction

As scientific innovations continue to progress, researchers have increasingly utilized lithium-ion battery (LIB) technology in areas such as electric vehicles and large-scale smart grids to tackle present energy and environmental issues [1,2]. However, conventional cathode materials of LIBs, like layered LiCoO_2_, spinel-type LiMn_2_O_4_, and olivine-type LiFePO_4_, have failed to address the requirements of electric vehicles owing to their capacity limitations [3,4]. Among the large number of potential cathode materials for LIBs, Li-rich Mn-based oxides (LRMs) have attracted considerable interest as advanced materials for next-generation LIBs, owing to their high theoretical capacity (≥250 mAh·g^−1^) and energy density surpassing 1000 Wh·kg^−1^ [5,6,7]. The substantial capacity of LRM arises from the combined involvement of oxygen anions and transition metal (TM) cations in charge balance. Nevertheless, LRMs are challenged by nanoscale structural destabilization during cycling, with irreversible O_2_ release, transition metal migration, and transformation of layered structure to spinel structure, leading to capacity decay, voltage decay, slow lithium-ion migration kinetics, and poor multiplicative performance. These nanoscale failure mechanisms have seriously hindered the commercialization of LRMs in power batteries [8,9,10].

To address the issues associated with LRMs, researchers have explored various modifications, including compositional modulation, doping, surface coating, and oxygen vacancy engineering [11,12]. Early studies found that oxygen vacancies (OVs) were one of the key factors influencing the structural changes and electrochemical behavior of LRMs [13]. At charging voltages above 4.5 V, lithium and oxygen in the Li_2_MnO_3_ component of LRMs are extracted as Li_2_O. The non-hybridized oxygen orbitals within the Li_2_-Li_3_ conformation induce oxygen instability, rendering it prone to oxidation. This process triggers the formation of O-O dimers, followed by O_2_ release, which generates OVs in the lattice [14]. These processes give rise to several challenges, such as a low initial Coulombic efficiency during the first charge–discharge cycle and the facilitation of irreversible TM ion migration to Li^+^ active sites, owing to the presence of residual OVs [15,16]. Experimental results indicated that actively introducing oxygen vacancies during synthesis effectively alleviated adverse effects from redox-active oxygen. The introduced oxygen vacancies’ reduced surface vacancies inhibited lattice oxygen release to prevent new vacancy formation, improved local Mn coordination to promote reversible TM migration, lowered charge-transfer resistance, and increased Li^+^ diffusion coefficients, thereby enhancing the material’s electrochemical performance [17,18,19,20,21]. Therefore, it is crucial to study the controllable construction of oxygen defects in LRMs and their mechanism of action in electrochemistry, and there is an urgent need to precisely regulate the oxygen activity and phase-interface stability through surface nanoengineering strategies.

**Figure 1:**
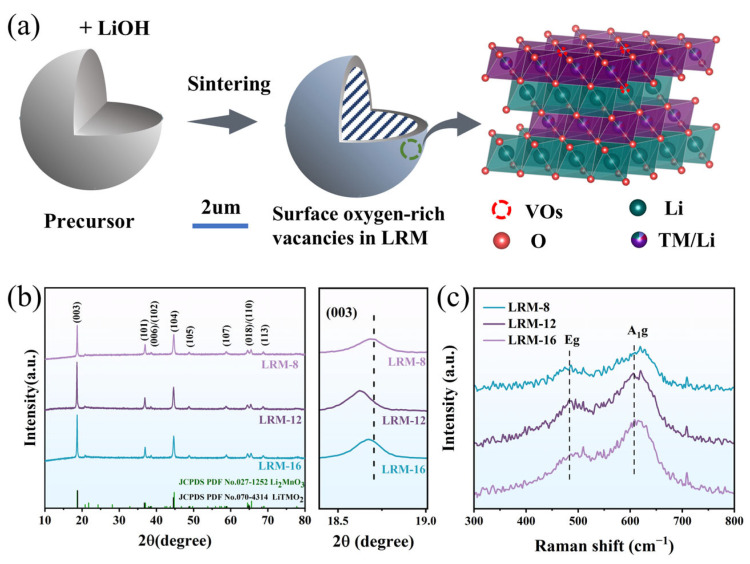
(**a**) Schematic diagram of the structure of the oxygen vacancies introduced by the LRM. (**b**) X-ray diffraction spectra of LRM-8, LRM-12, and LRM-16, with a localized magnification of the (003) crystal surface. (**c**) Raman spectra.

In this paper, a controllable oxygen vacancy engineering strategy has been established through a facile thermal treatment scheme to realize the Li-rich Mn-Based oxides’ (Li_1.2_Mn_0.54_Ni_0.13_Co_0.13_O_2_) surface reconstruction and structural stabilization, as shown in Figure 1a. OVs are precisely introduced via sintering time regulation, where reconstructed nanointerfaces accelerate lithium diffusion while optimized Mn coordination environments mitigate phase transitions. Benefiting from that, the treated Li_1.2_Mn_0.54_Ni_0.13_Co_0.13_O_2_ displays an initial reversible capacity of 247 mAh·g^−1^ at 0.2C, a capacity retention of 90.7%, and a voltage decay rate of 1.221 mV per cycle, indicating outstanding cycling stability and low voltage degradation. This research establishes a scalable defect control paradigm for nanostructured cathode materials by synergistically optimizing surface defects and bulk phase structure.

## 2. Experimental

### 2.1. Materials Synthesis

Mn_4_/_6_Ni_1_/_6_Co_1_/_6_CO_3_ carbonate precursors were synthesized by co-precipitation. First, MnSO_4_·4H_2_O (99%, Ron, Shanghai, China), NiSO_4_·6H_2_O (99%, Ron, Shanghai, China), and CoSO_4_·7H_2_O (99.5%, Ron, Shanghai, China) with a stoichiometry of 4:1:1 were dissolved in 50 mL of deionized water to prepare the transition metal salt solution. Meanwhile, ammonia (NH_3_·H_2_O, Xilong Science, Shenzhen, China) at a concentration of 2 mol/L was used as a complexing agent, as well as 50 mL of sodium carbonate (Na_2_CO_3_, 99.5%, Xilong Science, Shenzhen, China) at a concentration of 2 mol/L alkaline dissolved to induce precipitation. During the synthesis process, a transition metal salt solution, carbonate solution and ammonia solution were added dropwise at a constant rate to the reaction vessel at the same time, and the pH of the system was maintained at about 8, while the stirring rate was set at 600 rpm, and the reaction temperature was kept constant at 50 °C. When the solution dropwise addition was completed, stirring was continued for 2 h, and the reaction was allowed to stand for 8 h to sufficiently ripen the precipitate. In contrast, the stirring rate was set at 600 rpm, and the reaction temperature was kept constant at 50 °C. When the solution was completed, the stirring continued for 2 h, and the reaction was allowed to stand for 8 h to ripen the precipitate sufficiently. Subsequently, the resulting precipitate was separated by filtration and washed twice sequentially with deionized water and ethanol alternately and vacuumed at 80 °C for 10 h to obtain the precursor powder. The dried Mn_4_/_6_Ni_1_/_6_Co_1_/_6_CO_3_ was then mixed with 5% excess LiOH·H_2_O (99.5%, Xilong Science, Shenzhen, China), homogenized by ball milling, and subjected to a two-stage calcination process in oxygen atmosphere: initially heated to 500 °C at 3 °C/min with a 5 h hold, followed by sintering at 850 °C for 8, 12, or 16 h to produce the final Li_1.2_Mn_0.54_Ni_0.13_Co_0.13_O_2_ cathode materials (denoted as LRM-8, LRM-12, and LRM-16, based on sintering duration).

### 2.2. Characterization

The samples were subjected to Cu Kα radiation at a scan rate of 7°/min within the 2θ range of 5° to 80°, and their crystal structures were characterized using an X-ray diffractometer (X’Pert PRO-MPD, Nalytical, Almelo, The Netherlands). Rietveld refinement of the XRD data was performed with FullProf software. Raman spectroscopy (Thermo Nicolet 6700-NXR, Thermo Fisher Scientific, Waltham, MA, USA) was used to characterize the molecular composition and structure of the material, choosing the 532 nm laser wavelength. Morphological characterization was conducted via field-emission scanning electron microscopy (FE-SEM, Hitachi SU8600, Hitachi Corporation, Kyoto City, Japan) at 5 kV accelerating voltage. Transmission electron microscopy (TEM) analysis was carried out on a JEOL JEM-2100 (JEOL Japan Electronics Co., Ltd., Tokyo, Japan) microscope equipped with high-resolution TEM (HRTEM) capability. Oxygen vacancies were measured using electron paramagnetic resonance (EPR, Bruker ELEXSYS E500, Bruker, Berlin, Germany). The valence states and elemental composition of the samples were determined by X-ray photoelectron spectroscopy (XPS, ESCALAB 250Xi, Thermo Fisher Scientific, Waltham, MA, USA).

### 2.3. Electrochemical Measurements

The electrode materials consisted of 80 wt% active substance, 10 wt% carbon black, and 10 wt% poly(vinylidene fluoride) (PVDF), which were blended in N-methyl-2-pyrrolidone (NMP) to create a slurry. The mixture was then uniformly applied to the collector (aluminum foil) and dried under vacuum at 80 °C for 12 h. The area mass loading of each electrode was approximately 1.3 mg/cm^2^. The button cell was assembled in a glove box in the order of negative case (CR 2025), positive tab, diaphragm (Celgard 2400), negative (lithium tab) spacer (nickel mesh), negative case (CR 2025) with dropwise addition of the electrolyte (1.0 M LiPF6 in DMC:EC:EMC = 1:1:1) and was left to stand for 12 h. Using an electrochemical workstation, electrochemical impedance spectroscopy (EIS) was performed on the electrodes using an electrochemical workstation (CHI860D) in the frequency range of 100 kHz to 0.01 Hz. Cyclic voltammetry (CV) measurements were performed with an electrochemical workstation (CHI1000D) at a scan rate of 0.1 mV/s within a potential range of 2.0 to 4.8 V. The batteries were evaluated using the galvanostatic intermittent titration technique (GITT) and constant-current charge/discharge tests, conducted with a multichannel battery cycler Neware CT-4008T-5V (50 mA-164, Thermo Fisher Scientific, Waltham, MA, USA). Galvanostatic intermittent titration technique (GITT) and charge/discharge cycling were performed on a Neware CT-4008T-5V cycler (2.0–4.8 V, 25 °C) with 1C rate defined as 250 mA·g^−1^.

### 2.4. Density Functional Theory Calculations

The computational modeling of the Li_1.2_Mn_0.54_Ni_0.13_Co_0.13_O_2_ layered structure was performed within the density-functional theory (DFT) framework. The Vienna Ab-initio Simulation Package (VASP 5.3.x) was employed with the projector augmented wave (PAW) pseudopotential to describe electron–ion interactions. A 5 × 1 × 1 supercell was constructed, and oxygen vacancies were introduced by removing individual oxygen atoms. The Perdew–Burke–Ernzerhof (PBE) generalized gradient approximation (GGA) was selected for the exchange-correlation function due to its proven reliability in transition metal oxide systems. A plane-wave basis set with a cutoff energy of 500 eV was adopted and validated by convergence tests (energy fluctuation < 0.01 eV/atom) to ensure computational accuracy. Brillouin zone integration was carried out using a Γ-centered 2 × 10 × 2 k-point mesh to capture the anisotropic electronic density of states. Structural optimization was conducted via the conjugate gradient algorithm, where atomic positions were relaxed until residual forces fell below 0.01 eV/Å and total energy changes were less than 1 × 10^−5^ eV/atom. To address strong electron correlations in Mn(3d) and Co(3d) orbitals, Hubbard corrections within the DFT+U formalism were applied with U values of 4.5 eV (Mn) and 3.3 eV (Co).

## 3. Results and Discussion

Figure 1b presents the XRD patterns of LRM-8, LRM-12, and LRM-16. All major peaks were indexed to the layered α-NaFeO_2_-type structure (R-3m space group) with negligible impurity phases, confirming high crystallinity and phase purity. Additional weak reflections observed in the 20–25° 2θ range were assigned to the monoclinic Li_2_MnO_3_ phase (C2/m space group), demonstrating characteristic long-range Li/Mn ordering through superlattice arrangements. The (006)/(102) and (018)/(110) peak pairs of all samples were well-resolved, indicating that a highly ordered layered structure is exhibited by the material. The I(003)/I(104) ratios for all samples, which reflect the degree of cation mixing and structural disorder, were determined to exceed 1.2 (Figure 1b), suggesting minimal cation mixing [22]. The (003) crystallographic peak between 18.5° and 19° on the right side of Figure 1b is shown as a localized enlargement, the (003) peak of all samples shifted to lower angles to varying extents with changes in sintering time, which can be attributed to electrostatic interactions and lattice distortion, resulting in an increase in lattice spacing. Additionally, the structural characteristics of the synthesized cathode materials were further examined using Rietveld refinement (Figure S1), with the fitted results provided in Table 1 aligning with previous findings. The lattice parameters *a* and *c* were observed to increase with increasing heat treatment time. The c/a ratios for all samples exceeded 4.99, suggesting that each sample exhibited a well-defined hexagonal layered structure. Figure 1c presents the Raman spectra of the three samples, where peaks around 492 cm^−1^ and 607 cm^−1^ were attributed to the *E*g (O-TM-O bending) and *A*_1_g (TM-O stretching) vibrations in the LiTMO_2_ structure, respectively. The three weak peaks between 326 cm^−1^ and 442 cm^−1^ were assigned to Li-O phonon vibrations in the Li_2_MnO_3_ component [23]. Notably, the shoulder peak observed at 650 cm^−1^ in the LRM-12 sample was correlated with the Mn-O stretching vibrational mode in the surface spinel phase, resulting from atomic structure rearrangement induced by surface heat treatment [24].

**Figure 2:**
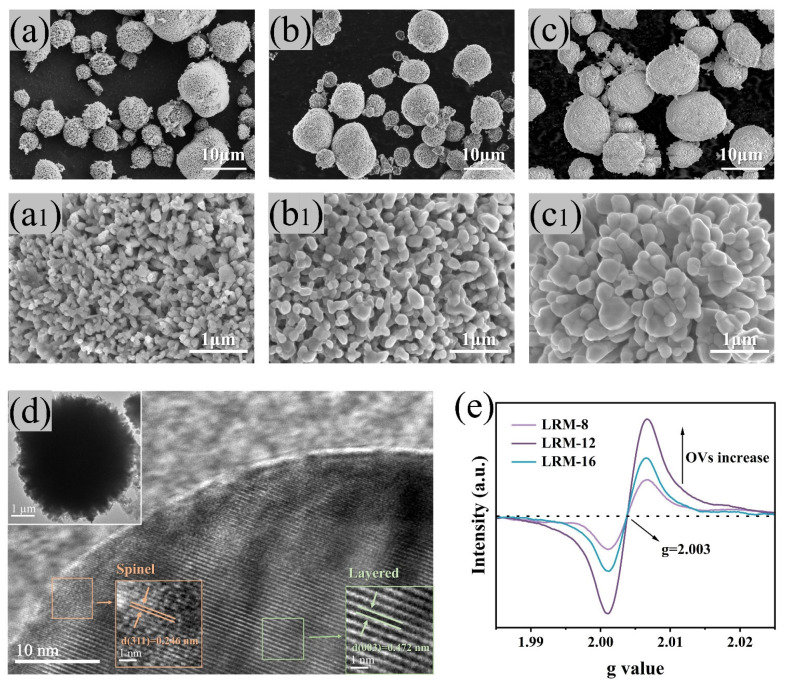
SEM images of cathode materials with different sintering times. (**a**) LRM-8, (**b**) LRM-12, (**c**) LRM-16; enlarged SEM images of (**a1**) LRM-8, (**b1**) LRM-12, (**c1**) LRM-16. (**d**) TEM images of LRM-12. (**e**) EPR spectra of LRM materials with different sintering times.

The impact of sintering time on the morphology and structure of LRM secondary particles (Figure 2a–c) and primary particles (Figure 2a1–c1) was investigated using SEM. The overall morphology of the carbonate precursor and its magnified view are shown in Figure S2. The LRM retained a spherical structure resembling that of the precursor. Examination of the primary particle morphology on the LRM surface revealed that sintering time strongly influenced grain growth. A few monodisperse irregular nanosheets remained on the LRM-8 surface, indicating the impact of sintering time on the morphology and structure of LRM secondary particles (Figure 2a–c) and primary particles (Figure 2a1–c1) was investigated using SEM. The overall morphology of the carbonate precursor and its magnified view are shown in Figure S2. The LRM retained a spherical structure resembling that of the precursor. Examination of the primary particle morphology on the LRM surface revealed that sintering time strongly influenced grain growth. A few monodisperse irregular nanosheets remained on the LRM-8 surface, indicating incomplete reaction during sintering. After 12 h of sintering, the particle contact changes from point contact to surface contact, the particle interfaces evolve and expand, the pores gradually shrink and deform, and the pores formed by CO_2_ release migrate to the particle interface and disappear [25]. At this stage, the particles became angular, and the primary particles exhibited a nearly octagonal structure, displaying a relatively uniform and narrow particle size distribution with diameters ranging from 100 to 200 nm. The LRM was observed to provide a large contact area for the electrolyte while structural integrity was enhanced. When the sintering time was increased to 16 h, the grain size was found to increase with uneven distribution. The pores were further shrunk, forming isolated closed pores that hindered complete electrolyte penetration into the material and extended the Li^+^ diffusion path. Elemental distribution and atomic percentages for LRM are shown in Figure S3a–c and Table S1. Ni, Co, Mn, and O were uniformly distributed with compositions aligned to theoretical values, confirming that the heat treatment did not alter the elemental homogeneity. The sample also exhibited a high degree of microstructural stability. The detailed microstructure of the LRM-12 samples was further examined using HRTEM, as shown in Figure 2d. The regions displayed clear lattice fringes, indicating a well-defined crystal structure. Fast Fourier Transform analysis revealed that the lattice spacing measured at 0.472 nm in the highlighted green area corresponded to the (003) crystal plane of the *C2/m* structure. Remarkably, in the orange region at the edge of the sample, (311) crystal facets with a lattice spacing of 0.246 nm were observed, matching the spinel phase. This observation further suggests that the high-concentration oxygen vacancy gradient induces surface structure reconstruction and spinel-like surface layer formation. EPR is widely employed to detect oxygen vacancies in metal oxide materials due to its ability to provide direct information about material defects. As shown in Figure 2e, all samples exhibited EPR signals at *g* ≈ 2.003, which were correlated with OVs [26]. The oxygen vacancy signal intensity first increased and then decreased with prolonged sintering time. During sintering, lattice oxygen stability was progressively reduced over time. Partial lattice oxygen was volatilized as Li_2_O, facilitating oxygen vacancy generation on the LRM surface. When the sintering time reached 16 h, partial grain surface melting occurred, accompanied by increased grain size and reduced specific surface area. Consequently, the maximum surface oxygen vacancy concentration was observed at 12 h [27]. This trend was consistent with the SEM analysis results.

**Figure 3:**
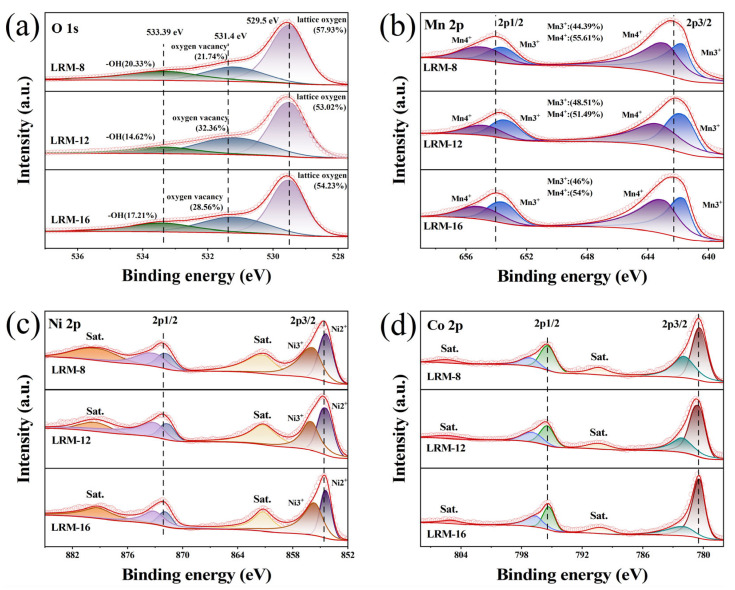
XPS spectra of LRM with different sintering times. (**a**) O 1s, (**b**) Mn 2p, (**c**) Ni 2p, (**d**) Co 2p.

XPS analysis was conducted to investigate the surface composition of LRM, as shown in Figure 3. In Figure 3a, the peak at 529.5 eV in the O 1s spectrum is attributed to lattice oxygen [28]. The peak at 531.4 eV is related to chemisorbed oxygen on oxygen vacancies, while the -OH signal at 533.39 eV may originate from adsorption with trace water molecules in the air during storage [29]. The introduction of OVs on the LRM surface is confirmed by the oxygen vacancy content, which is consistent with the EPR results. Figure 3b shows the Mn 2p spectra, where the signal peaks at 654.1 eV and 642.3 eV correspond to the Mn 2p1/2 and Mn 2p3/2 orbitals, respectively. The characteristic peaks of Mn^4+^ appeared at 642.83 eV and 654.83 eV, while the corresponding peaks of Mn^3+^ were located at 641.71 eV and 653.58 eV [30]. The lowest Mn^4+^ content (51.49%) and the highest Mn^3+^ content (48.51%) in LRM-12 are observed, suggesting that the treatment-induced reduction in Mn oxidation states facilitates the formation of active spinel phases on the material surface. In the Co 2p and Ni 2p spectra, shown in Figure 3c,d, no peak shifts were observed for any of the samples, suggesting that the oxidation states of Ni and Co remain unchanged. The Ni 2p XPS spectrum displayed characteristic photoelectron peaks at 872.1 eV (Ni 2p_1_/_2_) and 854.7 eV (Ni 2p_3_/_2_). Within the Ni 2p_3_/_2_ spectral region, two distinct chemical states were resolved at binding energies of 854.4 eV (Ni^2+^) and 855.8 eV (Ni^3+^) [31]. A spin-orbit splitting energy of 17.5 eV was measured between the Ni 2p_1_/_2_ and Ni 2p_3_/_2_ peaks, suggesting the predominant presence of Ni^2+^ species in the LRM compound [32]. The Co 2p XPS spectra were characterized by two distinct peaks located at 780.5 eV and 795.49 eV, corresponding to the Co 2p_3_/_2_ and Co 2p_1_/_2_ spin-orbit components, respectively. A spin-orbit splitting value of 14.99 eV was observed between these photoelectron signals, which suggested the predominant presence of Co^3+^ species in all three analyzed samples.

**Figure 4:**
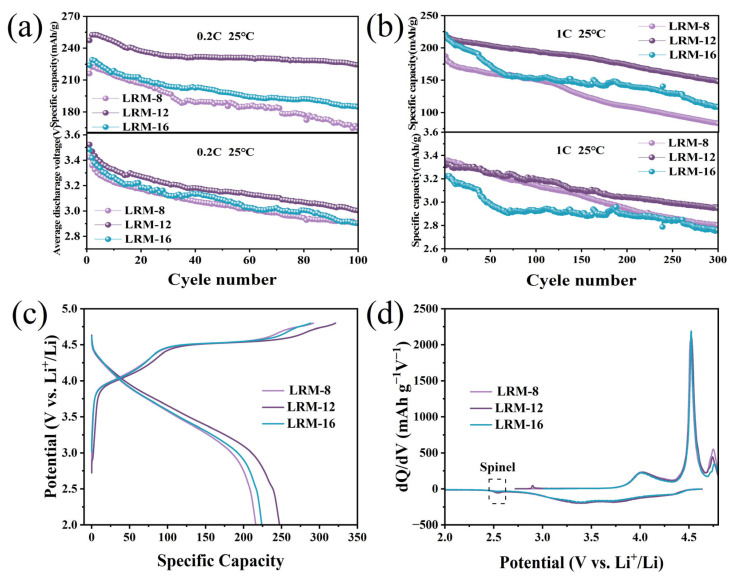
Cycling performance (upper panel) and average discharge voltage (lower panel) of LRM at different multiplication rates: (**a**) 0.2C, (**b**) 1C, (**c**) first charge–discharge curve of LRM at 0.2C, (**d**) differential capacity versus voltage plots.

A series of electrochemical tests were conducted to evaluate the influence of heat treatment duration on the electrochemical behavior of LRM. As illustrated in Figure 4a, the cyclic performance tests conducted at a 0.2C rate (1C = 250 mAh·g^−1^) revealed that LRM-12 exhibited the highest initial discharge specific capacity of 247 mAh·g^−1^, surpassing both LRM-8 (216 mAh·g^−1^) and LRM-16 (224 mAh·g^−1^). Furthermore, LRM-12 demonstrated superior cycling stability, with a retained capacity of 224 mAh·g^−1^ after 100 cycles, achieving a capacity retention rate of 90.7%. In comparison, significantly lower retained capacities of 164 and 185 mAh·g^−1^ were observed for LRM-8 and LRM-16 under identical cycling conditions, corresponding to retention rates of 75.9% and 82.6%, respectively. At a 1C rate, as shown in Figure 4b, the initial capacities of LRM-8, LRM-12, and LRM-16 were determined to be 187, 218 and 221 mAh·g^−1^, respectively. After 300 cycles, a capacity of 149 mAh·g^−1^ was retained by LRM-12, corresponding to a retention rate of 68.3%. In contrast, retention rates of 44% and 49.4% were observed for LRM-8 and LRM-16, respectively, after 300 cycles. This phenomenon is attributed to the insufficient reaction caused by short sintering time, which affects the structural stability of the material. Conversely, excessively long sintering time leads to increased grain size, which prolongs the Li^+^ diffusion path and degrades electrochemical performance. Additionally, a higher median voltage was exhibited by LRM-12. The median voltage decay of LRM-12 was measured as 1.2 mV/cycle during long-term cycling at 1C, which is lower than those of LRM-8 (1.83 mV/cycle) and LRM-16 (1.53 mV/cycle). It was confirmed that the introduction of OVs effectively suppressed structural phase transitions, leading to a reduction in voltage decay. As presented in Table S2, the electrochemical performance of Li-rich manganese-based oxides in this work is compared with those reported in previous studies, demonstrating significant advancement. The initial charge and discharge curves of the LRM materials at 0.2C are shown in Figure 4c. In the charging region below 4.5 V, the capacity contribution is primarily attributed to the charge compensation of Ni^2+^ and Co^3+^ in the active LiTMO_2_. The plateau around 4.5 V is related to the precipitation of Li^+^ in Li_2_MnO_3_ and the redox reaction of lattice oxygen. It is noteworthy that the discharge curve of LRM-12 shows a small insignificant gentle slope around 2.5 V due to the insertion of Li^+^ into the empty 16c octahedral position. This process is found to induce the reduction of Mn^4+^ to Mn^3+^, accompanied by a structural transition of the lithium manganese oxide spinel phase from cubic to tetragonal symmetry [33]. The differential capacity (dQ/dV) curve of LRM is displayed in Figure 4d. In the charging phase, a minor peak near 4 V is associated with the oxidation of Ni^2+^ to Ni^3+^ and Ni^4+^, as well as the oxidation of Co^3+^ to Co^4+^ within the LiTMO_2_ structure. The prominent oxidation peak observed at 4.5 V is linked to the activation process of Li_2_MnO_3_. The reduction peaks at 3.6 V and 4.7 V in the discharge phase correspond to the reduction process of LiTMO_2_. A prominent peak near 2.6 V is identified in LRM-12, which is assigned to the reduction of Mn^3+^ to Mn^4+^, thus validating the presence of the spinel phase in the material.

**Figure 5:**
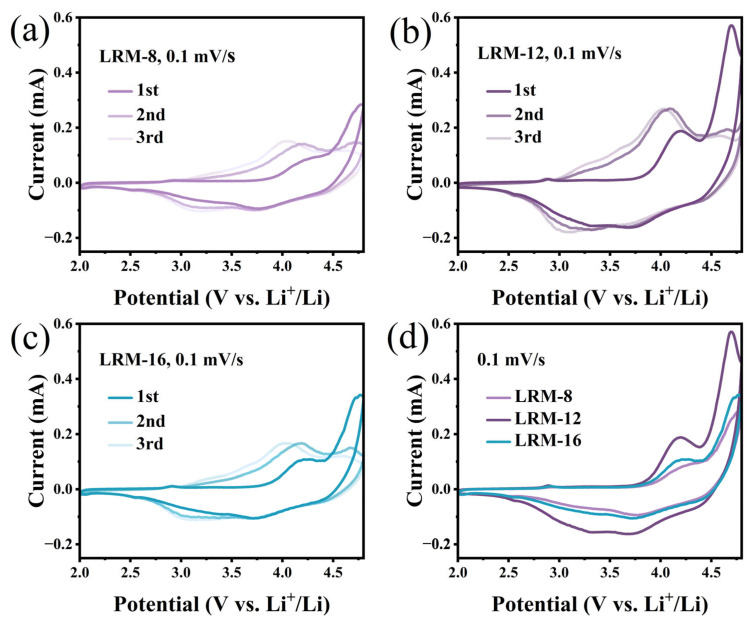
CV curves for the first three cycles of LRM. (**a**) LRM-8, (**b**) LRM-12, (**c**) LRM-16, (**d**) first-cycle CV curve of LRM.

The first three-turn CV curves of LRMs with different sintering times were obtained by cyclic voltammetry at a scan rate of 0.1 mV·s^−1^, as shown in Figure 5a–c. In the voltage range of 2–4.8 V, two pairs of redox peaks are exhibited in each curve. The initial peak located near 4.2 V is attributed to the oxidation of Ni^2+^ → Ni^3+/4+^ and the conversion of Co^3+^ → Co^4+^. The second peak, observed near 4.7 V, is linked to the activation of Li_2_MnO_3_ and the oxidation of oxygen. The peak observed at 3.2 V is assigned to the reduction of Ni^4+^ → Ni^3+/2^ and C^4+^ → Co^3+^. The peak around 3.7 V is attributed to Mn^4+^ → Mn^3+^ reduction. These reduction peaks then gradually move to lower potentials, indicating cation migration and structural phase changes. As shown in Figure 5d, a comparison of the first-cycle CV curves of the three samples reveals that LRM-12 exhibits a higher peak intensity at 4.7 V, suggesting enhanced oxygen release during the initial charging process [34].

**Figure 6:**
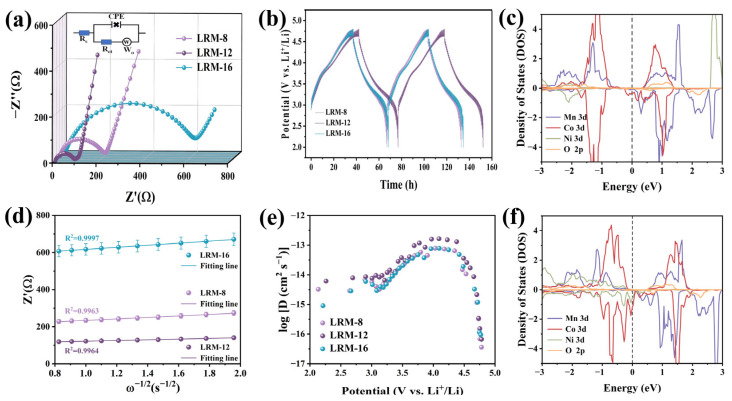
EIS curves (**a**) and linear fitting of Z′ versus ω^−1^/^2^ (**d**) for LRM. GITT curves (**b**) and calculated Li^+^ diffusion coefficients (**e**) for LRM. PDOS for LMR (**c**) and LMR-OV (**f**).

As shown in Figure 6a, the EIS spectra of LRM materials are presented, where each curve is characterized by a semicircular arc in the high-frequency region and a linear slope in the low-frequency region. The charge transfer resistance (R_ct_) value of LRM-12 was determined to be 81.55 Ω, which was significantly lower than those of LRM-8 (189.9 Ω) and LRM-16 (586.1 Ω). The Li^+^ diffusion coefficients (D_Li_^+^) for all samples were calculated from the corresponding slopes, with detailed values provided in Table S3 of the Supporting Information [35]. As shown in Figure 6d, the D_Li_^+^ are determined as 1.7 × 10^−14^, 7.8 × 10^−14^ and 8.9 × 10^−15^ cm^2^·s^−1^. These results demonstrate that stabilized lattice oxygen and increased surface oxygen vacancies effectively reduce the charge transfer resistance, whereas the surface spinel structure is responsible for enhancing the Li^+^ diffusion coefficient. The Li^+^ diffusion kinetics in LRM materials were further analyzed through GITT, with the calculation methodology detailed in the Supporting Information (Figure S4) [36]. Based on the experimental data, the lithium-ion diffusion profiles and corresponding D_Li_^+^ values are presented in Figure 6b,e, showing similar diffusion trends across all three samples. After two cycles, a prolonged lithium-ion diffusion time was observed for LRM-12 compared to LRM-8 and LRM-16, which exhibited higher D_Li_^+^ values. We performed to analysis of the electronic structures of the pristine LRM and its oxygen-deficient counterpart (LRM-OV). The atomic configurations of LRM before and after oxygen vacancy introduction are illustrated in Figure S5. The total density of states (TDOS) profiles for both LRM and LRM-OV, calculated through DFT, are presented in Figure S6. Figure 6c,f display the projected density of states (PDOS) for individual atoms in LRM and LRM-OV, respectively. The results demonstrate a notable increase in the electronic density of states at the Fermi level for LRM-OV, indicating enhanced electrical conductivity. Furthermore, the reduced bandgap near the Fermi level in LRM-OV provides additional evidence for the conductivity improvement induced by oxygen vacancies.

**Figure 7:**
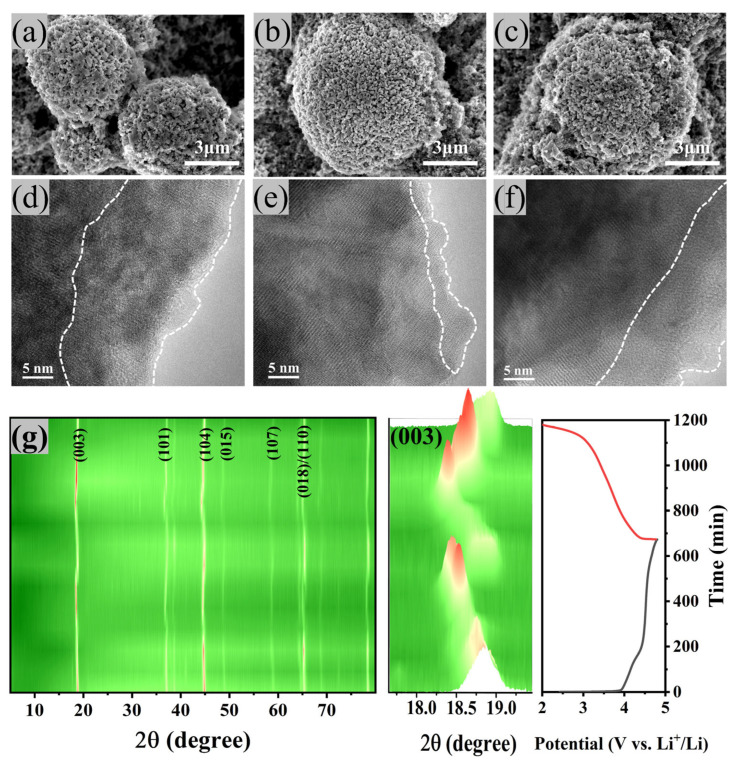
SEM images of LRM materials after cycling. (**a**) LRM-8, (**b**) LRM-12, (**c**) LRM-16. HRTEM images of LRM materials after cycling. (**d**) LRM-8, (**e**) LRM-12, (**f**) LRM-16. (**g**) Ex-suin XRD analysis of the first cycle of LRM-12.

To elucidate the role of OVs in structural evolution during cycling, morphological and crystalline transformations were systematically investigated through advanced characterization techniques. As shown in Figure 7a–c, post-cycling SEM images of the three samples after 100 cycles at 0.2C were obtained. Severe electrolyte-induced corrosion was observed on the LRM-8 surface, which was attributed to insufficient crystallinity leading to irreversible structural degradation. In contrast, the pronounced structural collapse was identified in LRM-16, primarily ascribed to continuous oxygen release and microdefect accumulation during prolonged cycling [37]. In contrast, the primary particle morphology and secondary particle architecture of LRM-12 were largely retained, indicating that OVs played a critical role in stabilizing the crystalline framework of the material. HRTEM analysis of the samples after 200 cycles was conducted, as illustrated in Figure 7d–f. Distinct surface reconstruction layers were observed in the near-surface regions of all LRM variants. LRM-8 and LRM-16 exhibited nearly complete phase transformation into mixed spinel-layered phases, accompanied by localized rock-salt structures with thicknesses of ~17 nm and ~14 nm, respectively. In contrast, LRM-12 retained its original layered structure in the bulk phase, featuring a thin spinel surface layer (~5 nm). These findings demonstrate that the introduction of OVs effectively suppressed structural phase transitions, thereby mitigating voltage and capacity degradation. The structural evolution of LRM-12 during the first charge/discharge cycle was investigated via Ex-suin XRD within a voltage window of 2.0–4.8 V, as shown in Figure 7g. A detailed analysis of the (003) crystal plane revealed a progressive shift of the (003) peak toward lower angles during charging (2.0–4.5 V), which was attributed to lattice parameter *c* expansion and enhanced electrostatic repulsion between oxygen anions during Li^+^ extraction. Between 4.5 and 4.8 V, lattice parameter *c* contraction was observed, primarily caused by oxygen loss and transition metal migration into Li layers [38,39,40]. Upon discharging to 2.0 V, the peak position was nearly restored to its original state, indicating that LRM-12 exhibits superior structural stability.

## 4. Conclusions

In summary, oxygen vacancies were introduced into Li_1.2_Mn_0.54_Co_0.13_Ni_0.13_O_2_ through a controlled thermal treatment process. The presence of surface OVs was confirmed by electron EPR and XPS analyses. OV concentration exhibited an initial increase followed by a decrease with prolonged sintering duration. SEM characterization revealed that extended sintering progressively destabilized lattice oxygen, while partial lattice oxygen volatilized as Li_2_O, generating oxygen-deficient regions on LRM surfaces. When the sintering duration reached 16 h, a localized interfacial fusion between primary particles was observed, accompanied by grain coarsening and reduced specific surface area, which explained the maximum OV concentration at 12 h. TEM analysis demonstrated that high-concentration OV gradients induced surface reconstruction and the formation of a spinel-like interfacial layer, creating additional Li^+^ diffusion pathways. The modified Mn coordination environment further promoted reversible cation migration and delayed phase transitions, collectively enhancing structural stability and mitigating voltage decay. The optimized LRM delivered a discharge capacity of 247 mAh·g^−1^ at 0.2C with 90.7% capacity retention after 100 cycles. Even under 1C cycling, a low voltage decay rate of 1.221 mV/cycle was maintained over 300 cycles, demonstrating exceptional cycling stability.

## Figures and Tables

**Table 1:** Lattice Parameters and the Ratio of I(003)/I(104) of Samples.

Sample	a (Å)	c (Å)	c/a	I_(003)_/I_(104)_
LRM-8	2.8501	14.2468	4.9987	1.26
LRM-12	2.8512	14.2897	5.0118	1.65
LRM-16	2.8486	14.2490	5.0021	1.56

## Data Availability

The original contributions presented in this study are included in the article/Supplementary Material. Further inquiries can be directed to the corresponding author.

## Supplementary Materials

The following supporting information can be downloaded at: https://www.mdpi.com/article/10.3390/ma18112537/s1, Figure S1: Rietveld refinement of the XRD spectrum of the LRM sample.; Figure S2: SEM image of the precursor sample; Figure S3: EDS pattern of LRM sample. (a) LRM-8, (b) LRM-12, (c) LRM-16; Figure S4: The diffusion coefficients of Li^+^ ions were evaluated by GITT test at 0.1 C in the voltage range; Figure S5: The atomic configurations of LRM before and after oxygen vacancy introduction. (a) LRM, (b) LRM-VO; Figure S6: TDOS curves for LRM and LRM-VO; Table S1: EDS-Mapping results of element amount of O/Mn/Co/Ni for LRM samples; Table S2: Comparison of electrochemical properties of Li-rich Mn-based oxides for Lithium-ion batteries; Table S3: EIS fitting data for LRM samples. Refs. [21,41,42,43,44,45,46,47,48] can be found in Supplementary Materials.
