# Supplementary material for: Surface Oxygen Vacancy Modulation of Nanostructured Li-Rich Mn-Based Oxides for Lithium-Ion Batteries
Source: Materials (Basel). 2025 May 28;18(11):2537. doi: 10.3390/ma18112537 (PMC12155591; doi:10.3390/ma18112537)
Supplement: Supplementary file 1 [file materials-18-02537-s001.zip › materials-3615002-supplementary.pdf]

Supporting Information

Surface Oxygen Vacancy Modulation of Nanostructured Li-rich Mn-Based Oxides for Lithium Ion Batteries

Jinxia Nong, Xiayan Zhao, Fangan Liang, Shengkun Jia, Zhengguang Zou*.

College of Materials Science and Engineering, Guilin University of Technology, Guilin 541004, China.

* Corresponding authors: Prof. Zhengguang Zou (zouzgglut@163.com)

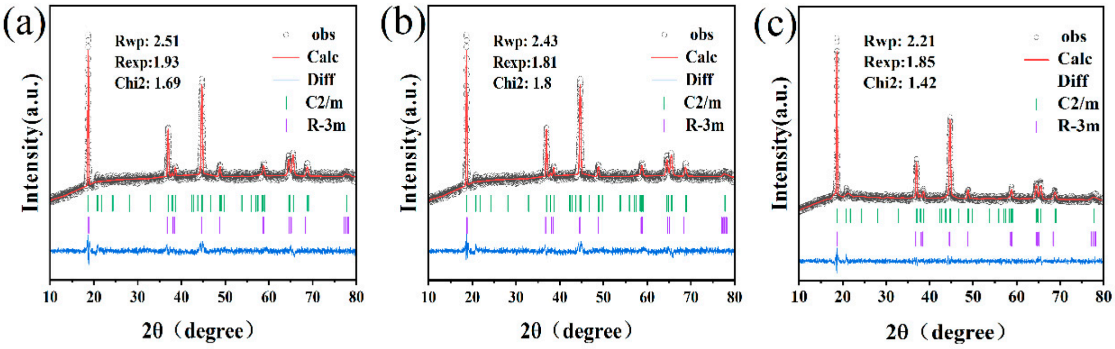

Figure S1. Rietveld refinement of the XRD spectrum of the LRM sample.

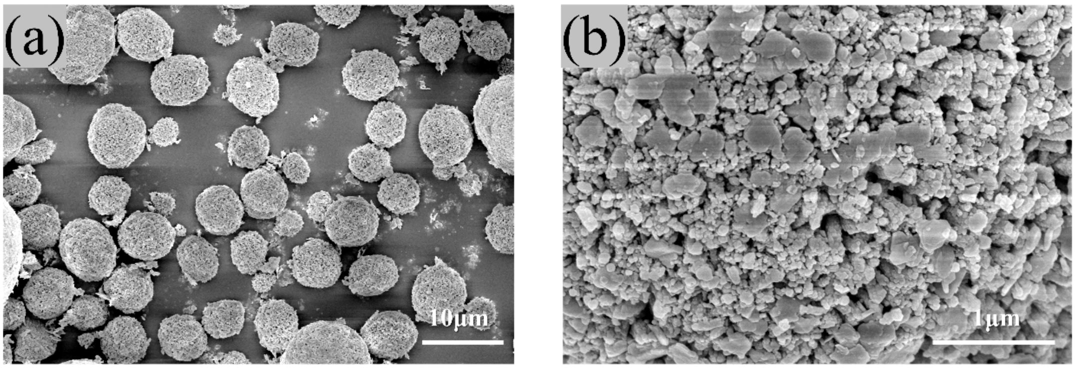

Figure S2. SEM image of the precursor sample.

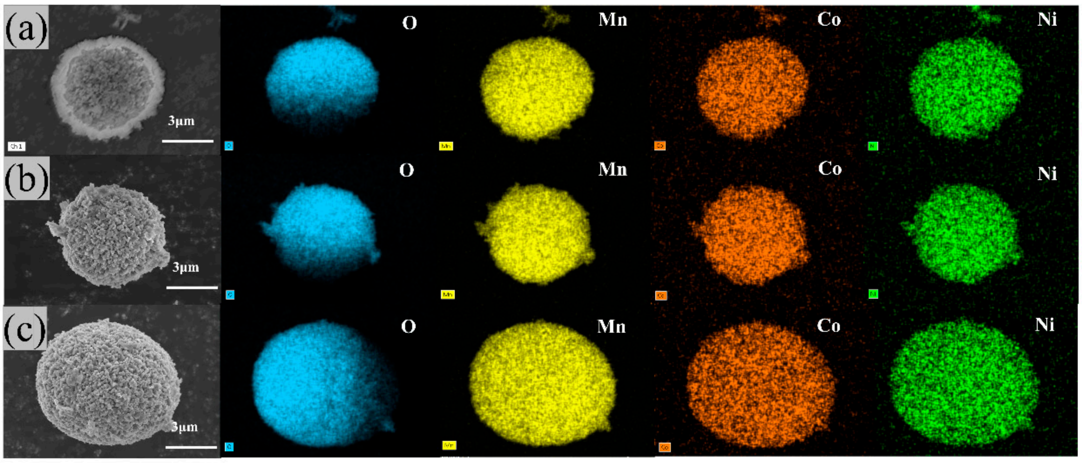

Figure S3. EDS pattern of LRM sample. (a) LRM-8, (b) LRM-12, (c) LRM-16.

The diffusion coefficients of Li^+ ions were evaluated by GITT test at 0.1 C. Li^+ diffusion coefficient (D_{Li+}) can be calculated by Eq:

$$D_{Li+}=\frac{4}{\Pi\tau}\left(\frac{mV}{MS}\right)^2\left(\frac{\triangle Es}{\triangle E\tau}\right)^2 \quad (\tau \ll L_2/D_{Li+}) \qquad [S1]$$

Where m is the mass of the active substance in the working electrode, M and V represent the molar mass and molar volume of the LRM, L is the thickness of the electrode, S is the active surface area of the electrode, and τ is the duration of the applied current. Eτ and Es are the transient voltage change during a single titration current flux and the steady state voltage change after the relaxation period, respectively.

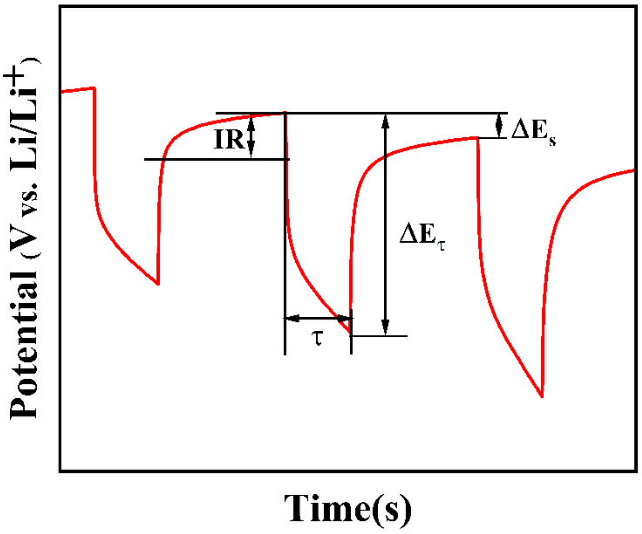

Figure S4. The diffusion coefficients of Li^+ ions were evaluated by GITT test at 0.1 C in the voltage range.

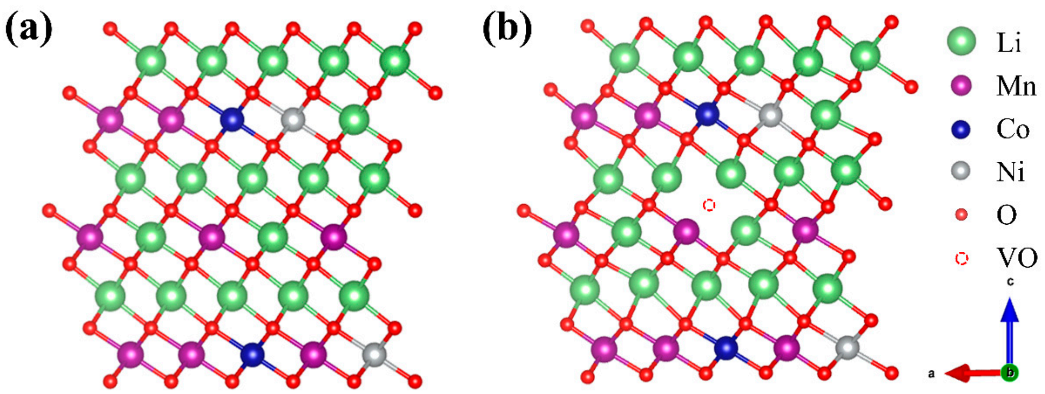

Figure S5. The atomic configurations of LRM before and after oxygen vacancy introduction. (a)LRM, (b)LRM-VO.

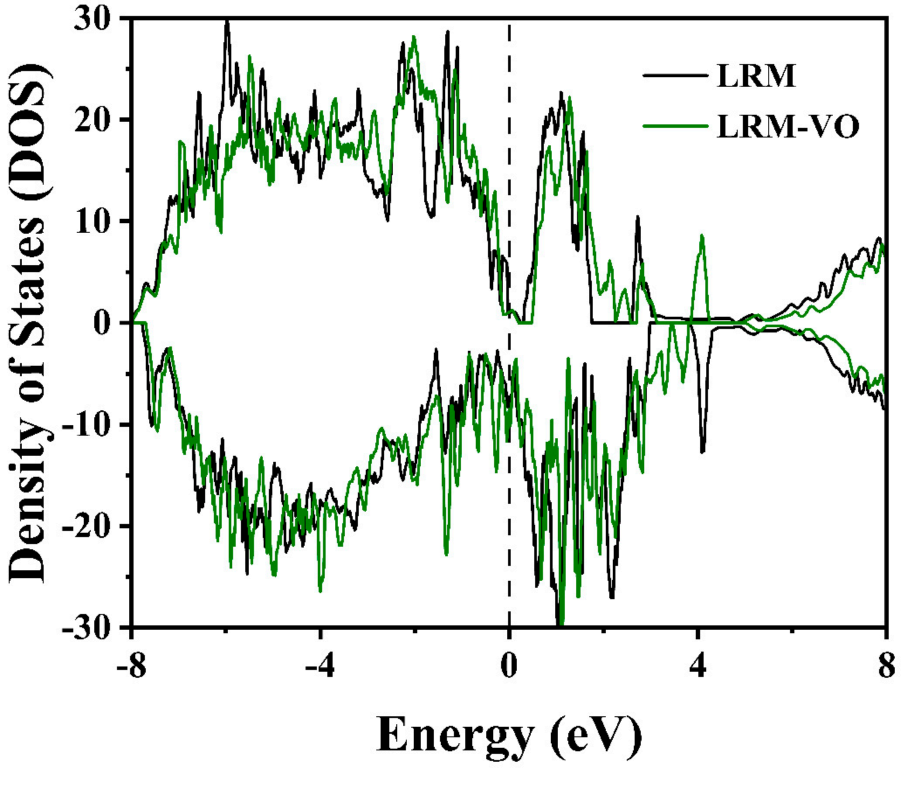

Figure S6. TDOS curves for LRM and LRM-VO

Table S1. EDS-Mapping results of element amount of O/Mn/Co/Ni for LRM samples.

Sample	Atom/%			
	O	Mn	Co	Ni
LRM-8	79.97	13.41	3.3	3.32
LRM-12	76.24	15.26	3.87	4.04
LRM-16	78.73	14.3	3.54	3.43

Table S2. Comparison of electrochemical properties of Li-rich Mn-based oxides for Lithium-ion batteries.

Cathode material	Discharge capacity (mA h g^{-1})	Parameters for electrochemical properties	Ref.
$Li_{1.2}Mn_{0.6}Ni_{0.2}O_2$	204 (0.1c)	87.4%(n=100)	[2]
$Li_{1.2}Mn_{0.56}Ni_{0.17}Co_{0.07}O_2$	275(0.1C)	63%(n=200)	[3]
$Li_{1.2}Mn_{0.54}Co_{0.127}Yb_{0.003}Ni_{0.13}O_2$	267.2(0.2C)	84.69%(n =100)	[4]
$Li_{1.2}Co_{0.13}Ni_{0.13}Mn_{0.5}O_2$	198.8(0.2C)	78.3%(n=100)	[5]
$Li[Li_{0.25}Ni_{0.1}Co_{0.05}Mn_{0.6}]O_2$	224(0.5C)	72.9%(n=100）	[6]
$0.5Li_2MnO_3 \cdot 0.5LiNi_{1/3}Co_{1/3}Mn_{1/3}O_2$	262(0.1C)	83.6%(n=100)	[7]
$Li_{1.2}Ni_{0.32}Co_{0.04}Mn_{0.44}O_2$	193.2(1C)	79.3%(n=80)	[8]
$Li_{1.2}Ni_{0.13}Co_{0.13}Mn_{0.54}O_2$	219(1C)	71%(n=200)	[9]
$Li_{1.2}Ni_{0.13}Co_{0.13}Mn_{0.54}O_2$	247.2(0.2C)	90.7%(n=100)	This work
$Li_{1.2}Ni_{0.13}Co_{0.13}Mn_{0.54}O_2$	218.1 (1C)	67%(n=300)	This work

Table S3. EIS fitting data for LRM samples.

Sample	Rs(Ω)	Rct(Ω)	δ/Ωcm^{2}g$^{-1/2}$	D_{Li+}(cm^{2}g^{-1})
LRM-8	3.42	189.9	39.95	1.725×10^{-14}
LRM-12	6.56	81.55	18.79	7.796×10^{-14}
LRM-16	3.77	586.1	55.74	8.866×10^{-15}

References

[1] Wang, Q.; Yao, M.; Zhu, A.; Wang, Q.; Wu, H.; Zhang, Y. Semi-Metallic Superionic Layers Suppressing Voltage Fading of Li-Rich Layered Oxide Towards Superior-Stable Li-Ion Batteries. ANGEW CHEM INT EDIT 2023, 62, e202309049 https://doi.org/10.1002/anie.202309049.

[2] Liu, S.; Yan, X.; Li, P.; Tian, X.; Li, S.; Teng, F.; Luo, S.-h. Structural and enhanced electrochemical performance of Co-free lithium-rich layered manganese-based $Li_{1.2}Mn_{0.6}Ni_{0.2}O_2$ cathodes via Na-doping at Li site for lithium-ion batteries. Mater 2024, 28, 101027. https://doi.org/10.1016/j.mtsust.2024.101027.

[3] Wang, Z.; Yin, Y.; He, G.; Zhao, H.; Bai, Y. Improving the long-term electrochemical performances of Li-rich cathode material by encapsulating a three-in-one nanolayer. Nanoscale 2023, 15, 588-598. https://doi.org/10.1039/d2nr04074c.

[4] Li, Q.; Wang, H.; Wang, G.; Xia, F.; Zeng, W.; Peng, H.; Ma, G.; Guo, A.; Dong, R.; Wu, J. Stabilized Li-Rich Layered Oxide Cathode by a Spontaneously Formed Yb and Oxygen-Vacancy Rich Layer on the Surface. Small 2023, 20, 2307419. https://doi.org/10.1002/smll.202307419.

[5] Zhao, T.; Liu, Z.; Gu, Q.; Zhang, X.; Jin, X.; Xie, S.; Liu, S. New exploration of water-soluble lithium polyacrylate/xanthan gum composite binder for Li-rich Mn-based cathode materials. Chem. Phys. Lett 2024, 841, 141182. https://doi.org/10.1016/j.cplett.2024.141182.

[6] Sun, Y.; Huang, J.; Zhang, H.; Zhang, L.; Wang, D. Controllable Synthesis and Surface Modifications of a Metastable O2-Type Li-Rich Cathode Material. Crystals 2023, 13, 1154-1165. https://doi.org/10.3390/cryst13081154.

[7] Abdel-Ghany, A. E.; El-Tawil, R. S.; Hashem, A. M.; Mauger, A.; Julien, C. M. Integrated Lithium-Rich $yLi_2MnO_3 \cdot (1-y)LiNi_{1/3}Co_{1/3}Mn_{1/3}O_2$ Layered Cathode Nanomaterials for Lithium-Ion Batteries. Int J Mol Sci 2025, 26, 1346-1376. https://doi.org/10.3390/ijms26031346.

[8] Lin, F.; Wen, J.; Zhu, H.; Tang, Y.; Li, Z.; Li, T.; Wang, Y.; Chen, Z. Highly activated oxygen redox enabling large-capacity Li-rich layered manganese-based oxide cathodes. PCCP 2023, 25, 15271-15278. https://doi.org/10.1039/d3cp01935g.

[9] Wang, W.; Cheng, W.; Huang, Y.; Wang, Y.; Wei, Y.; Liu, Q. One-step construction of oxygen vacancies and coating to improve lithium storage performance of Li-rich layered oxides. Appl. Surf. Sci 2022, 605. https://doi.org/10.1016/j.apsusc.2022.154819.
